# Development and validity and reliability testing of a questionnaire on life and death education cognition and selection preference of cancer patients

**DOI:** 10.3389/fonc.2026.1822600

**Published:** 2026-10-27

**Authors:** Runqin Huang, Xingyu Zhang, Wei Chen, Yonghong Li, Jing Li

**Affiliations:** 1 Cerebrovascular Department, Affiliated Hospital of Zunyi Medical University, Zunyi, Guizhou, China; 2 Nursing Department, Affiliated Hospital of Zunyi Medical University, Zunyi, Guizhou, China; 3 Department of Oncology, The Third Affiliated Hospital of Zunyi Medical University, Zunyi, Guizhou, China; 4 Department of Oncology, The Second Affiliated Hospital of Zunyi Medical University, Zunyi, Guizhou, China

**Keywords:** cancer, cognition, life and death education, questionnaire, selection preference

## Abstract

**Objective:**

To develop a questionnaire on life and death education cognition and selection preference of cancer patients and test its reliability and validity.

**Methods:**

This study is a methodological research aimed at developing a questionnaire and conducting psychometric evaluation on it. The 5W1H method is used as the theoretical framework for the preference dimensions of life and death education. The initial questionnaire was developed through literature analysis, qualitative interviews, and delphi expert consultation. A questionnaire survey was conducted on 300 cancer patients hospitalized in the oncology department of a general hospital in Zunyi from March 2022 to July 2022 to test the reliability and validity of the questionnaire.

**Results:**

The questionnaire of life and death education cognition and selection preference of cancer patients included 25 items in 3 dimensions(life and death education cognition, life and death education attitude, life and death education selection preference). Validity and reliability of graded score items. The item-level content validity index ranged from 0.86 to 1.00;the scale-level content validity index of all consistency was 0.90;the scale-level average content validity index was 0.986. The Cronbach’s α coefficient, half reliability and retest reliability of the overall scale were 0.954,0.892,and 0.853,respectively. and the Cronbach’s α coefficients of each dimension were 0.85, 0.947, and 0.943, respectively.

**Conclusion:**

The questionnaire of life and death education cognition and selection preference of cancer patients has good reliability and validity, and has the value of clinical application. It can be used as an auxiliary tool to evaluate the life and death education cognition and life and death education needs of cancer patients.

**Clinical trial registration:**

https://www.chictr.org.cn/, identifier ChiCTR2600134780.

## 1 Introduction

Cancer is one of the main causes threatening human health, and the burden of cancer incidence and mortality is rapidly increasing worldwide (1). According to the Global Burden of Cancer Report, the incidence of cancer in China is increasing rapidly, with an average annual growth rate of more than 2% (2). The China Cancer Report points out that new cases of cancer in China account for about 1/4 of new cases of cancer worldwide (14.09 million), and an average of 7 people are diagnosed with cancer every minute (3). Cancer is the disease with the highest morbidity and mortality in China (4), and has become an important chronic disease endangering the life safety and health of Chinese people (5). Cancer is a progressive deterioration of the disease, its high recurrence rate and mortality make patients in a state of anxiety, fear and depression for a long time, seriously affecting the daily life of patients and leading to a serious decline in their quality of life (6–8). Life and death education is to provide overall care at the psychological, social and spiritual levels for cancer patients (9), help identify different types of anxiety and fear in cancer patients, and give management measures for patients who identify anxiety and fear, while assisting family members to provide strong psychological support, spiritual support and companionship for patients, aiming to guide patients and family members to scientifically recognize and treat death and accept death calmly (10–13). However, due to insufficient cultural background and public cognition, cancer patients’ life and death education cognition is lacking in China, and there is a lack of scientific and applicable life and death education cognition and demand assessment tools for cancer patients, and life and death education-related research is difficult to carry out. Accurate assessment of the needs of life and death education for cancer patients is conducive to medical staff to carry out targeted life and death education intervention and psychological guidance, and improve the quality of life of patients. Therefore, this study developed a localized questionnaire on life and death education cognition and selection preference of cancer patients in China and tested its reliability and validity in order to provide a tool reference for assessing cancer life and death education cognition and needs.

## 2 Study methods

### 2.1 English back translation

The original questionnaire was developed in Chinese. For the purpose of English publication, two independent bilingual translators (one with a nursing background and one without a nursing background) performed forward translation of the Chinese version into English. A third translator, blinded to the original document, back-translated the English version into Chinese. The research team compared the back-translated Chinese version with the original version, discussed and resolved discrepancies. This process ensured semantic equivalence. The final English version of the questionnaire is shown in **Table 1**.

**Table 1:** Questionnaire on cognition and selection preference of life and death education for cancer patients.

Dimension	Item
A.Life and Death Education Cognition	
cognition	A1: Have you heard of life and death education?
	A2: Do you know what life and death education is?
	A3: Do you know what hospice care is?
B.Life and death education attitude	
attitude	B1: Would you like to talk about life and death?
	B2: Would you like to talk about your end-of-life wishes?
	B3: Are you willing to receive knowledge about life and death education?
	B4: Would you like to receive more information about your death?
	B5: Would you like to receive life and death education to help you establish a correct and positive outlook on life and death?
C.Life and Death Education Choice Preference	
Why (necessity)	C1: Do you think it is necessary to discuss life and death?
	C2: Do you think you have an urgent need for life-and-death education?
	C3: Do you think it is necessary to educate cancer patients about life and death?
	C4: Do you think it is necessary to educate the family members of cancer patients about life and death?
What (content)	C5: Do you want to understand the process of individual end-of-life?
	C6: Do you want to know what life and death education is?
	C7: Do you want to understand the purpose and significance of birth-death education?
	C8: Do you want to know how to relieve pain, fatigue and other symptoms?
	C9: Do you want to understand how psychological and mental distress is regulated in relation to illness and death?
	C10: Do you want to know how to prepare for death?
	C11: Would you like to know more about hospice care?
	C12: Do you want to understand the ethical and legal issues associated with death?
When (timing)	C13: At what stage of disease progression do you think life and death education should be conducted?
	C14: How often do you think life and death education should be conducted?
Where (location)	C15: Where do you think life and death education is appropriate?
Who (provider)	C16: Who do you think can carry out life and death education?
How (method)	C17: Which way do you want to learn about life and death education?

### 2.2 Prepare draft scale

#### 2.2.1 Theoretical framework

Taking 5W1H method as the theoretical framework of choice preference of life and death education, 5W1H method analyzes all-round and multi-dimensional problems from six aspects: Why (necessity),What (content), When (timing), Where (location), Who (provider), and How (method), and then puts forward solutions to problems, the core of which is multi-dimensional analysis problems and multi-faceted problems (14). To comprehensively understand the needs of life and death education for cancer patients and combine with the current situation of life and death education for cancer patients, the first draft of the questionnaire sets up items from six aspects: the content, necessity, time, place, personnel, and methods of life and death education, including one initial dimension of life and death education selection preference.

#### 2.2.2 Establishing entry pools

##### 2.2.2.1 Literature analysis identify entry pools

With “death education”、 “life and death education” and “education of death” as keywords, Chinese and English databases such as CNKI Academic Journal Database, Wanfang Data Knowledge Service Platform, Chinese Biomedical Literature Service System, VIP Information Resource System, Pubmed, Web of Science, and OVID LWW Journals were searched from database establishment to August 2021. Through literature analysis and collation, the preliminary proposed questionnaire contained life and death education choice preference questionnaire items.

##### 2.2.2.2 Supplementary item pool for qualitative interviews

A total of 17 cancer patients were selected for qualitative interviews using the purposive sampling method in strict accordance with the inclusion and exclusion criteria of the study. According to the study objective and literature review, the outline of the visit included the following eight open questions: ① Have you heard about life and death education before? ② Have you received any courses or lectures related to life and death education? ③ What content do you want to know about life-and-death education? ④ Do you think it is necessary to conduct life and death education? ⑤ When do you think life and death education should begin? ⑥ Where do you think life-and-death education is more appropriate? ⑦ Who do you want to provide life and death education to? ⑧ What ways do you want to understand life and death education? The interview data were collated and analyzed by the research team using Colaizzi’s phenomenological seven-step analysis method and Nvivo qualitative research and analysis software within 24 hours after the end of the interview. Combined with the interview results, the item pool was revised and supplemented to increase the cognitive dimension of the life and death education. Form a pool of entries containing 2 dimensions and 25 entries.

#### 2.2.3 Performing expert consultation

##### 2.2.3.1 Selection of expert consultants

Fourteen experts were selected to revise the first draft of the questionnaire between December 2021 and February 2022. Inclusion criteria: (1) Have worked in the above-mentioned fields for ≥10 years; (2) Hold a bachelor’s degree or above; (3) Hold an associate senior professional title or above; (4) Be willing to complete two rounds of consultation. Exclusion criteria: Unable to return the questionnaire on time or withdraw from the research process. The final expert panel consists of 14 experts, which meets the sample size recommended for Delphi method research. One expert withdrew during the expert consultation process, and the final number of experts was 14.

##### 2.2.3.2 Formulation of letter query form and entry screening method

The consultation form included four parts: letter inquiry description, project content consultation form, expert basic information questionnaire, and familiarity and judgment basis questionnaire. ① Letter inquiry description: Introduced the purpose of this study, the contents to be completed by experts, and the precautions for filling in. ② Item Content Consultation Form: Likert 5-level scoring method was used for importance scoring of consultation content, and 1 ~ 5 points were counted from “very unimportant” to “very important”, respectively. ③ The questionnaire form of basic information of experts collected the general data of experts, such as name, age, educational background, professional title, research field, and years of professional engagement. ④ Familiarity and judgment basis questionnaire: This was used to judge the familiarity of experts with the consultation content and judgment basis. E-mail and WeChat were used to distribute and return the questionnaires. The duration of each round of letter inquiry was two weeks, and the interval was two weeks. The item screening method was to retain the items with a mean value of importance assignment > 3.50 points, and a coefficient of variation < 0.25. Simultaneously, the items were modified and adjusted according to expert opinions to increase the dimensions of life and death education attitude and form the first draft of the questionnaire containing 25 items in three dimensions.

### 2.3 Pre-investigation

According to the inclusion and exclusion criteria of this study, 30 hospitalized cancer patients were investigated using convenience sampling in March 2022. The results showed that the patients could understand the content of the questionnaire, fill in the questionnaire appropriately (15–20 minutes), and finally did not modify the questionnaire.

### 2.4 Study design

The study was divided into two phases: (1) questionnaire development through literature review, qualitative interviews, and two rounds of Delphi expert consultation; (2) Psychometric evaluation of the questionnaire through cross-sectional surveys.

### 2.5 Cross-sectional surveys

#### 2.5.1 Respondents

A convenience sampling method was used to select 300 cancer patients hospitalized in the oncology department of a general hospital from March 2022 to June 2022 as the survey subjects. Sample size estimation: According to the principle that the sample size should be 5–10 times the number of questionnaire items, and considering 10%-20% of invalid questionnaires, the final sample size of the questionnaire survey in this study was 300 cases (15). Inclusion criteria 1. Hospitalized patients with pathologically confirmed cancer; 2. Awareness of their condition; 3. Aged 18 years old or older, normal intelligence, no communication barriers, with reading and expression ability; 4. Participants accepted the topic of life and death education, voluntarily participated in this study, and signed an informed consent form. Exclusion criteria:1. Patients with mental disorders, senile dementia, and cognitive impairment; 2. Patients with unstable or sharp deterioration of their condition; 3. Patients are often disgusted by the topic of death.

#### 2.5.2 Investigation tools

##### 2.5.2.1 General information questionnaire

The following data were collected by the investigator: including gender, age, residence, education level, ethnicity, occupation, marital status, religious belief, educational background, monthly family income, source of cost, disease diagnosis, clinical stage, treatment method, and duration of illness.

##### 2.5.2.2 The questionnaire on cognition and selection preference of life and death education for cancer patients

The questionnaire consists of 3 dimensions with a total of 25 items, including cognition (3 items), attitude (5 items), and selection preference (17 items). All items are scored by the 5-level Likert scoring method. For the cognition dimension, scores range from 1 to 5 from “very unfamiliar” to “very familiar”, with a total score of 3 to 15 points. For the attitude dimension, scores range from 1 to 5 from “very unwilling” to “very willing”, with a total score of 5 to 25 points. The selection preference dimension contains 5 multiple-choice items that are not scored. For the remaining items, scores range from 1 to 5 from “very undesirable” to “very desirable”, with a total score of 12 to 60 points. A higher score indicates a better level of cognition, attitude or selection preference regarding life-and-death education among cancer patients. **Table 1** provides a full mapping of each item to the 5W1H framework.

#### 2.5.3 Quality control

The subjects were included in the study in strict compliance with the inclusion and exclusion criteria to ensure the homogeneity of the samples. Before the start of the survey, the investigators received unified training to avoid artificial deviations. If the patient had difficulty in filling in the questionnaire, the investigators explained it to the patient. The completeness of the questionnaire content was checked on the spot, and if there were missing items or missing items, the questionnaire was immediately supplemented and verified with the patient. The data entry was checked by two persons to ensure the accuracy of the data entry, and the data entry was reviewed by two persons to ensure the accuracy of the data.

### 2.6 Statistical methods

SPSS 26.0 software was used for statistical analysis of the data. Enumeration data were described by frequency and constituent ratio, and measurement data in accordance with normal distribution were described by mean ± standard deviation. Experts’ enthusiasm is evaluated by expert’s positive coefficient (effective recovery rate of each letter inquiry), experts’ authority is evaluated by expert’s authority coefficient (average value of expert’s familiarity coefficient and expert’s judgment basis coefficient), and expert’s opinion coordination is evaluated by the Kendall harmony coefficient. Statistical significance was set at P < 0.05.

### 2.7 Ethical considerations

This study was conducted in accordance with the Helsinki Declaration and approved by the Ethics Committee of the Affiliated Hospital of Zunyi Medical University (Approval Number: KLLY-2021-082). All participants provided written informed consent prior to enrollment. They were informed of the purpose, procedures, and voluntary nature of the study, and assured that they had the right to withdraw from the study at any time without affecting their medical care. During the data entry and analysis process, personal identifiers were removed to ensure confidentiality.

## 3 Results

### 3.1 Expert consultation results

A total of 14 experts were interviewed by letter, including 1 male and 13 females, aged 36-70 (50.64 ± 8.72) years; 5 doctors, 4 masters, 5 undergraduate; 9 senior and 5 sub-senior; 6 nursing management/nursing education, 8 oncology nursing/hospice care; and working hours (30.57 ± 10.18) years. Fifteen and 14 questionnaires were collected from the first and second rounds of consultation, respectively, and the positive coefficients of the two rounds of consultation experts were 100% and 93.33%, respectively, indicating that the two rounds of consultation experts showed high enthusiasm. The authoritative coefficients of the two rounds of expert correspondence were 0.90 and 0.89, respectively, indicating a high degree of expert authority. The Kendall coordination coefficients were 0.160 (χ2 = 57.697, P < 0.001) and 0.212 (χ2 = 70.770, P < 0.001), indicating that the communication inquiry results were well-coordinated.

The importance scores of the items in the first round of expert consultation ranged from 3.53 to 4.87, and the coefficient of variation ranged from 0.07 to 0.44. A total of 12 experts made suggestions involving the modification/deletion, addition and merging of items on the scale. Three experts suggested combining the entries “you know what is hospice care”, “you know what is end-of-life care”, and “you know palliative care and palliative nursing” to “you know what is hospice care”. Experts recommend adding the entry “You want to understand the process of ending an individual’s life”. Experts recommend removing the entry, “You think it is necessary to implement universal life and death education.” Two experts suggested removing “you wanted to understand the meaning of death”. Experts recommend removing the entry “You want to understand the nature of death”. Experts recommend removing the entry “You want to know how to handle test-related issues”. Experts recommended modifying the entry “You want to understand hospice care and communication skills” to “You want to understand hospice care”. Experts recommended modifying the entry “You want to know how to relieve pain in chronic pain” to “You want to know how to relieve symptoms such as pain and fatigue”. Experts recommended modifying the entry “At what age do you think life and death education should be carried out?” to “At what stage do you think life and death education should be carried out?”. Experts recommend increasing cancer patients’ attitude toward life and death education in first-level items. The above-mentioned expert recommendations were adopted. According to the first round of expert consultation and revision comments, combined with the results of the topic group study and discussion, the expert letter questionnaire was revised and returned to the expert 2 weeks later for the second round of expert letters. Feedback of letter inquiry results: The importance score of the items in the second round of expert consultation was 4.50 - 5.00 points, and the coefficient of variation was 0.00 - 0.17. Experts suggest modifying the item “you are willing to talk to others about life and death” to “you are willing to talk about life and death” The remaining experts put forward no comments on revising the item. The resulting questionnaire contained three dimensions and 25 items. Details are provided in **Table 2**.

**Table 2:** Expert importance score results and coefficient of variation for each entry in round 2 letter inquiry.

Item	Full score rate(%)	Importance score ( $\bar{\text{X}}$± S)	Coefficient of variation
A.Life and Death Education Cognition	85.71	4.86 ± 0.36	0.08
A1: Have you heard of life and death education?	50.00	4.50 ± 0.52	0.12
A2: Do you know what life and death education is?	71.43	4.71 ± 0.47	0.10
A3: Do you know what hospice care is?	57.14	4.50 ± 0.65	0.14
B.Life and death education attitude	71.43	4.71 ± 0.47	0.10
B1: Would you like to talk about life and death?	64.29	4.64 ± 0.50	0.11
B2: Would you like to talk about your end-of-life wishes?	64.29	4.57 ± 0.65	0.14
B3: Are you willing to receive knowledge about life and death education?	100.00	5.00 ± 0.00	0.00
B4: Would you like to receive more information about your death?	64.29	4.57 ± 0.65	0.14
B5: Would you like to receive life and death education to help you establish a correct and positive outlook on life and death?	64.29	4.57 ± 0.65	0.14
C.Life and Death Education Choice Preference	92.86	4.93 ± 0.27	0.05
C1: Do you think it is necessary to discuss life and death?	71.43	4.71 ± 0.47	0.10
C2: Do you think you have an urgent need for life-and-death education?	78.57	4.79 ± 0.43	0.09
C3: Do you think it is necessary to educate cancer patients about life and death?	100.00	5.00 ± 0.00	0.00
C4: Do you think it is necessary to educate the family members of cancer patients about life and death?	78.57	4.79 ± 0.43	0.09
C5: Do you want to understand the process of individual end-of-life?	57.14	4.50 ± 0.65	0.14
C6: Do you want to know what life and death education is?	85.71	4.86 ± 0.36	0.07
C7: Do you want to understand the purpose and significance of birth-death education?	57.14	4.50 ± 0.76	0.17
C8: Do you want to know how to relieve pain, fatigue and other symptoms?	100.00	5.00 ± 0.00	0.00
C9: Do you want to understand how psychological and mental distress is regulated in relation to illness and death?	78.57	4.79 ± 0.43	0.09
C10: Do you want to know how to prepare for death?	71.43	4.71 ± 0.47	0.10
C11: Would you like to know more about hospice care?	57.14	4.50 ± 0.65	0.14
C12: Do you want to understand the ethical and legal issues associated with death?	57.14	4.50 ± 0.65	0.14
C13: At what stage of disease progression do you think life and death education should be conducted?	100.00	5.00 ± 0.00	0.00
C14: How often do you think life and death education should be conducted?	100.00	5.00 ± 0.00	0.00
C15: Where do you think life and death education is appropriate?	100.00	5.00 ± 0.00	0.00
C16: Who do you think can carry out life and death education?	100.00	5.00 ± 0.00	0.00
C17: Which way do you want to learn about life and death education?	100.00	5.00 ± 0.00	0.00

### 3.2 General information of respondents

A total of 300 cancer patients were included in this study, and tumor types were as follows: head and neck tumors in 118 patients (39.33%), thoracic tumors in 106 patients (35.33%), abdominal tumors in 73 patients (24.33%), and other tumors in 3 patients (1.00%). Gender: 174 males (58.00%) and 126 females (42.00%); age: (53.90 ± 11.74) y. Education level: 248 (82.67%) were junior high school or below, and 52 (17.33%) were senior high school or above. Marital status: 284 married (94.67%), 9 unmarried (3%), and 7 divorced/widowed (2.33%). Average monthly income: < 3000 RMB 250 (83.33%), ≥ 3000 RMB 50 (16.67%). Payment method of medical costs: 253 cases (84.33%) of resident medical insurance, 45 cases (15.00%) of employee medical insurance, and 2 cases (0.67%) of self-pay patients. Residence: 136 (45.33%) in towns and 164 (54.67%) in rural areas.

### 3.3 Reliability and validity analysis

#### 3.3.1 Construct validity

Exploratory factor analysis was used to determine the structural validity of the questionnaire. The Bartlett’s spherical test for the 20 items of the questionnaire scale showed that the KMO value was 0.935, and theχ²of Bartlett’s spherical test was 5727.208, P<0.01, indicating that the questionnaire in this study is suitable for factor analysis. Principal component analysis was used to extract factors with eigenvalues >1 as the standard. Finally, 3 common factors were extracted, and the cumulative variance contribution rate was 72.148%. The factor loading matrix of each factor is shown in **Table 3**.

**Table 3:** Factor loadings of each item in the questionnaire.

item	Factor 1 (cognitive dimension of life and death education)	Factor 2 (attitude toward life and death education)	Factor 3 (dimension of preference for life and death education choices)
A2	.919	.137	.092
A3	.878	.079	.057
A1	.816	.117	.028
B3	.150	.864	.296
B4	.112	.850	.313
B5	.109	.849	.338
B1	.163	.818	.297
B2	.176	.756	.263
C1	.112	.798	.388
C3	.114	.726	.367
C4	.084	.702	.390
C2	-.152	.551	.381
C8	.136	.145	.763
C9	.101	.244	.742
C7	.050	.536	.720
C6	.029	.525	.718
C11	.021	.488	.689
C12	-.051	.382	.684
C10	.065	.425	.669
C5	.092	.536	.636

#### 3.3.2 Content validity

Content validity is an important indicator of the quality of a questionnaire (12). Expert scoring results were used to calculate the item-level CVI (I-CVI) at the item level and the scale-level CVI (S-CVI) at the scale level. In this study, seven experts from the nursing management, surgical nursing, oncology nursing, and hospice nursing fields were selected to test the content validity of the questionnaire. The results showed that the questionnaire I-CVI ranged from 0.86 to 1.00, the consistent SCVI was 0.90, and the average SCVI was 0.986, indicating that the content validity of this questionnaire at the item level was good.

#### 3.3.3 Reliability analysis

The overall Cronbach’s α coefficient of the life and death education cognition and selection preference questionnaire for cancer patients was 0.954, and the Cronbach’s α coefficients of each dimension were 0.850, 0.947, and 0.943, respectively; the half-reliability of the total questionnaire was 0.892, and the half-reliability of each dimension was 0.715, 0.882, and 0.903, respectively; the test-retest reliability of the total questionnaire was 0.853, and the test-retest reliability of each dimension was 0.836, 0.852, and 0.790, respectively.

## 4 Discuss

### 4.1 Good scientificity of questionnaire

In this study, the 5W1H method was used as the theoretical framework, and the first draft of the questionnaire was formed by consulting the relevant literature on the current situation of life and death education for cancer patients at home and abroad and combining qualitative interviews. Fourteen experts in the relevant fields were screened for two rounds of Delphi expert correspondence. The experts included those from nursing management, nursing education, oncology nursing, and hospice care fields, and most of them had a postgraduate degree or above, with a working life of 11–41 years, and had solid research experience and clinical experience in their respective fields, which could provide professional and authoritative guidance for this study. The positive coefficients of the two rounds of consultation experts were 100% and 93.33%, respectively, and the enthusiasm of the experts for this study was high. Kendall ‘s coordination coefficients were 0.160 and 0.212, respectively, indicating that the experts’ authority was high and coordination was good. After two rounds of expert consultation, the preliminarily formed questionnaire was revised and improved, and the item content was determined after discussion by the research group. Finally, a formal questionnaire with 25 items in three dimensions was formed. In this study, the questionnaire production process was strict, cautious, scientific, and standardized. The reliability and validity of the questionnaire were good, and it could better reflect the cognition and selection preference of life and death education for cancer patients, laying a foundation for carrying out life and death education for cancer patients at a later stage.

### 4.2 Questionnaire has high clinical application value

In 2021, the Plan for the Development of the National Aging Undertakings and the Elderly Care Service System issued by the state clearly states that medical and health institutions shall be promoted to carry out hospice care services in accordance with the principle of “fully informed and voluntary selection” and vigorously carry out public life and death education (16). Further clarify the importance of carrying out and implementing life and death education in social and medical institutions. When cancer patients fail to respond to active treatment, most of them experience loss of emotional control, death anxiety, death fear and other psychological (17). However, in China, due to the traditional view of good life and evil death, traditional view of filial piety, religion, psychological cognition of patients and family factors, imperfect hospice care services in hospitals, insufficient cognition of life and death education for medical staff, imperfect construction of death education institutions and organizations, and imperfect relevant legal mechanisms, the popularization and application of life and death education in clinical practice are restricted (8), and medical workers lack sufficient communication skills and ability to discuss topics related to death with patients and their families. Although patients and their families feel anxious and fearful about death, there is no reasonable way and opportunity to relax the pain in their hearts, so they choose to speak quietly, resulting in the life and death education needs of cancer patients failing to be timely detected and met. Currently, there is a lack of qualitative research on life and death education for cancer patients in China. Therefore, this study used a qualitative interview form to communicate face-to-face with patients to understand their cognition, attitudes, and demand for life and death education. Based on this, a questionnaire on life and death education cognition and selection preference for cancer patients was constructed, which has practical significance for assisting clinical medical workers to understand the cognition, attitude, and demand of life and death education for cancer patients, carrying out life and death education for cancer patients, and improving the quality of life of cancer patients.

### 4.3 The questionnaire has a good application prospect

This study aimed to understand the cognition of death education and the needs of cancer patients at many aspects and different levels of death education, to improve and optimize the death education model in China, improve the quality of life of cancer patients to meet the real needs of cancer patients at different stages, and protect the dignity of cancer patients at the end of life.

In this study, through two rounds of authoritative expert consultation in the professional field, after analysis, discussion, and modification by the research group, a questionnaire consisting of 25 items in three dimensions was integrated, and the expert authoritative coefficient and coordination coefficient were analyzed, showing that the expert consultation method in this study was credible. After the final questionnaire was generated, 300 valid questionnaires were obtained through the questionnaire, and the analysis results showed that the questionnaire had good validity and reliability. A larger sample of investigation studies could be conducted in the future to analyze the multiple factors affecting the death education of cancer patients and carry out targeted improvement in order to improve the quality of life of cancer patients.

## 5 Conclusion

In summary, the questionnaire developed in this study has scientific and clinical application value and can be used to evaluate the life and death education cognition, attitude, and life and death education selection preference of cancer patients. However, this questionnaire also has the shortcomings of a single-center cross-sectional survey, sample size representativeness, and lack of cross-disciplinary cooperation. In the future, multi-center and large-sample mixed studies should be conducted to test the clinical practicability of the questionnaire and further promote the development of life and death education for cancer patients in China.

### 5.1 Implications for nursing and health policy

This study’s standardized assessment methods can considerably enhance the accuracy of nursing practice and humanistic care, allowing nurses to scientifically assess patient cognition and preferences, direct communication, and design tailored care plans. For health policies, the questionnaire data can provide a key empirical basis for promoting the pre-established medical care plan (ACP), optimizing the allocation of hospice care resources and health insurance payment reform, promoting policies that are more in line with patients’ actual needs, and promoting the improvement of the end-of-life care system.

## Data Availability

The original contributions presented in the study are included in the article/supplementary material. Further inquiries can be directed to the corresponding author.
